# Advances in tissue optical clearing for 3D imaging in large animal

**DOI:** 10.1007/s12200-025-00162-6

**Published:** 2025-08-18

**Authors:** Yating Deng, Jianyi Xu, Tingting Yu, Dan Zhu

**Affiliations:** 1 MOE Key Laboratory for Biomedical Photonics, Wuhan National Laboratory for Optoelectronics-Advanced Biomedical Imaging Facility, Huazhong University of Science and Technology, Wuhan, 430074 China; 2 Optics Valley Laboratory, Wuhan, 430074 China

**Keywords:** Tissue optical clearing, Optical imaging, Large animal, Biological tissues

## Abstract

Three-dimensional reconstruction of tissue architecture is crucial for biomedical research. Tissue optical clearing technology overcomes light scattering limitations in biological tissues, providing an essential tool for high-resolution three-dimensional imaging. Given the high degree of similarity between large model animals (e.g., pigs, non-human primates) and humans in terms of anatomical structure, physiologic function, and disease mechanisms, the application of this technology in these models holds significant value for biomedical research. While well-established tissue clearing protocols exist for tissue sections, whole organs, and even entire bodies in rodents, scaling up to large animal specimens presents substantial challenges due to dimensional effects and compositional variations. This review systematically examines the methodological translation from rodent to large animals, particularly on species-specific differences in brain architecture and parenchymal organ composition that critically impact clearing efficiency. We comprehensively summarize recent applications in large animals, focusing on representative areas including neural circuit mapping, sensory organ imaging, and other related research domains, while proposing optimization strategies to overcome cross-species compatibility barriers. We hope this review will serve as a valuable reference for advancing tissue optical clearing applications in large-animal biomedical research.

**Graphical Abstract:**

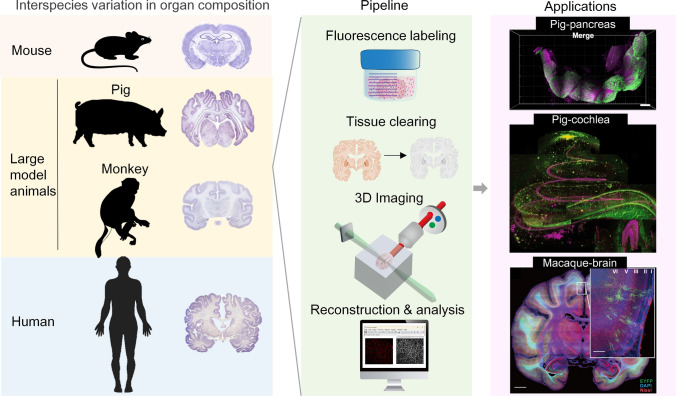

## Introduction

Deciphering three-dimensional (3D) structural information of biological tissues is a cornerstone for understanding organ development and disease mechanisms [1, 2]. While electron microscopy offers nanometer-scale resolution, its limited field of view hinders macroscopic tissue reconstruction [3, 4]. Conversely, clinical imaging modalities (MRI/CT, magnetic resonance imaging/computed tomography) provide whole-organ coverage but lack sufficient cellular/subcellular analysis resolution [5]. Optical microscopy theoretically bridges this gap [6, 7]; confocal [8], two-photon [9], and light-sheet microscopy [10] all enable 3D tissue reconstruction. However, inherent light scattering in biological tissues restricts effective imaging depth to hundreds of micrometers, severely limiting high-resolution 3D visualization of intact organs [7, 11]. The emergence of tissue optical clearing technology has revolutionized this field [12]. This technology significantly enhances light penetration depth by modulating RI (refractive index) homogeneity in biological tissues, enabling high-resolution 3D imaging at whole-organ scales [13, 14]. The methodology has evolved from mouse brain slices to whole-body clearing specifically in murine models [15], establishing three principal approaches: hydrophobic, hydrophilic, and hydrogel-embedding systems [16]. Notably, these advancements involve more than simple volumetric scaling—transitioning from brain slices to whole organs required solutions for penetration kinetics, while whole-body clearing presented challenges in heterogeneous organ co-clearing [17]. This “scale-method co-evolution” paradigm has progressively expanded clearing applications, providing novel tools for structural analysis in large animals. Despite these advancements, applying clearing techniques to large model animals, which is critical for biomedical research, encounters unique challenges due to interspecies biological divergence.

Large model animals are indispensable in biomedical research due to their anatomical and physiologic fidelity to humans [18–20]. For instance, non-human primates exhibit not only gyrencephalic brain morphology but also cognitive and behavioral parallels to humans [21], while porcine models replicate human cardiovascular and metabolic traits with high accuracy [22]. However, when applying tissue clearing to these species, a paradoxical “scale-comparable but method-incompatible” dilemma emerges: despite comparable physical dimensions, tissue heterogeneity often renders standard protocols ineffective. Current research predominantly adapts rodent-derived methods through parameter adjustments, lacking a dedicated clearing framework for large animals. This “scale-comparable but method-incompatible” dilemma represents the core challenge in large animal clearing applications. While existing reviews have summarized the advances of tissue optical clearing in specific fields (e.g., rodent neurobiology or human pathology), systematic discussions on its application to large model animals remain limited. This review examines the methodological progression of tissue clearing techniques at different scales in rodents, with particular attention to the challenges of adapting these methods for porcine and non-human primate tissues. We analyze how species-specific tissue properties—including heterogeneity, scaling kinetics, and structural variations—affect clearing performance in different organ systems, and summarize current applications in large animal research. By evaluating these limitations and discussing potential optimization strategies, this work aims to provide practical references for researchers working on cross-species tissue optical clearing applications.

## Physical principles and chemical strategies of tissue optical clearing

**Fig. 1:**
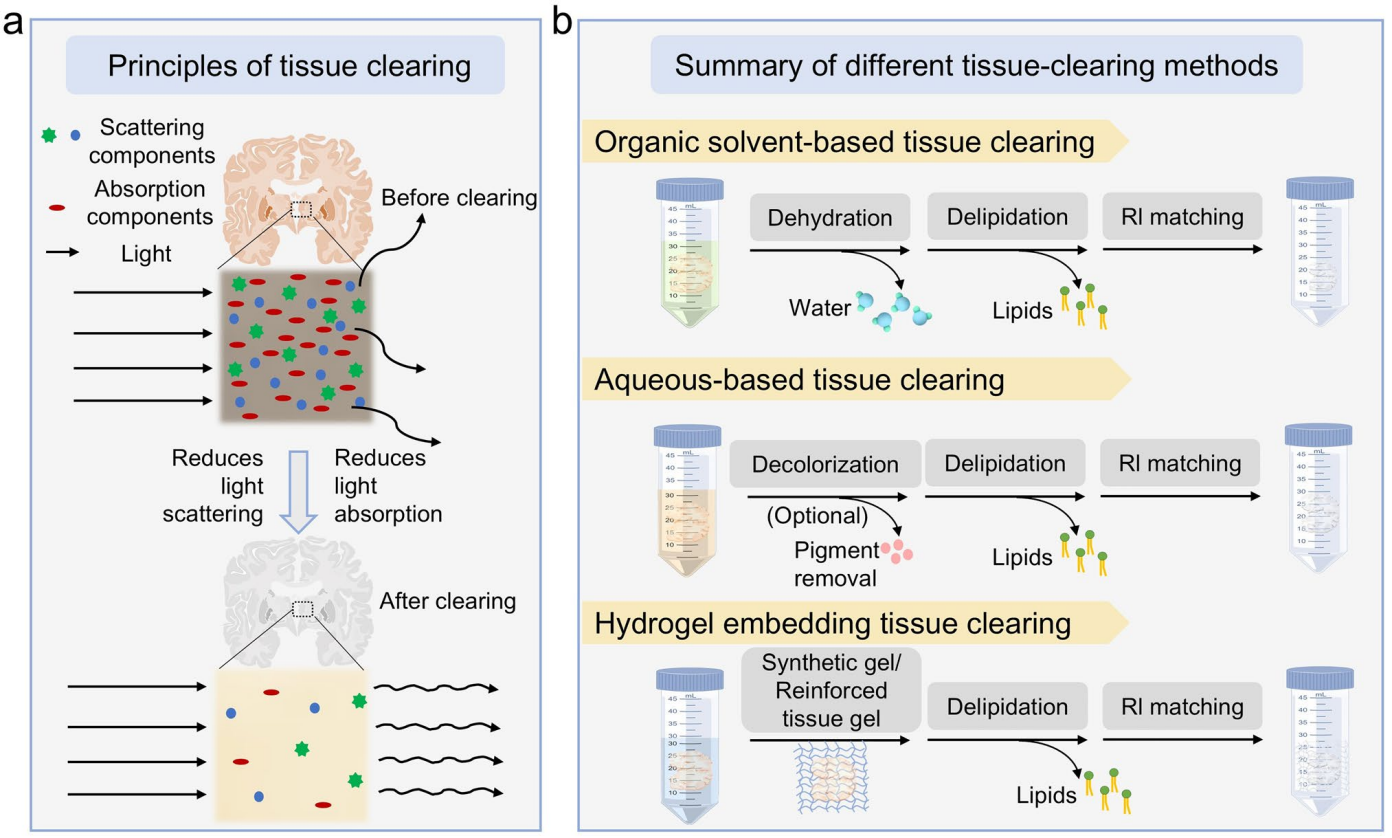
Principles and classification of tissue clearing techniques. **a** Tissue optical clearing is achieved by removing light-scattering and light-absorbing components, followed by RI matching using specialized media. Slices of brain adapted from © Wikimedia. **b** Comparative workflow of the three major clearing methodologies: organic solvent-based, aqueous-based, and hydrogel-embedding-based approaches

The opacity of biological tissues primarily originates from their intrinsic structural heterogeneity and RI mismatches among different components [23]. Within tissues, the disparity in refractive indices between various constituents (proteins, lipids, water, and minerals) leads to significant light scattering [24, 25]. Furthermore, microscopic structures such as collagen fibers and cellular membranes create numerous scattering interfaces, ultimately rendering the tissue opaque [26]. The fundamental objective of tissue optical clearing technology is to reduce RI heterogeneity through physical or chemical means, thereby enabling efficient light penetration through tissues (Fig. 1a) [27]. The underlying principles can be categorized into two main strategies [13]: the first involves removing or replacing highly scattering components while introducing RI-matching media to achieve uniform RI distribution throughout the tissue. Specific approaches include delipidation, dehydration, decolorization, and RI matching. The alternative strategy focuses on physically modifying the tissue’s microstructure to decrease the density of scattering interfaces, primarily through hydrogel-embedding techniques and expansion microscopy.

As illustrated in Fig. 1b, existing tissue optical clearing methods are typically classified into three categories based on their processing strategies: organic solvent-based, aqueous-based, and hydrogel-embedding. This classification reflects the diverse chemical approaches developed to address the challenge of tissue opacity while accommodating different experimental requirements and sample characteristics.

**Fig. 2:**
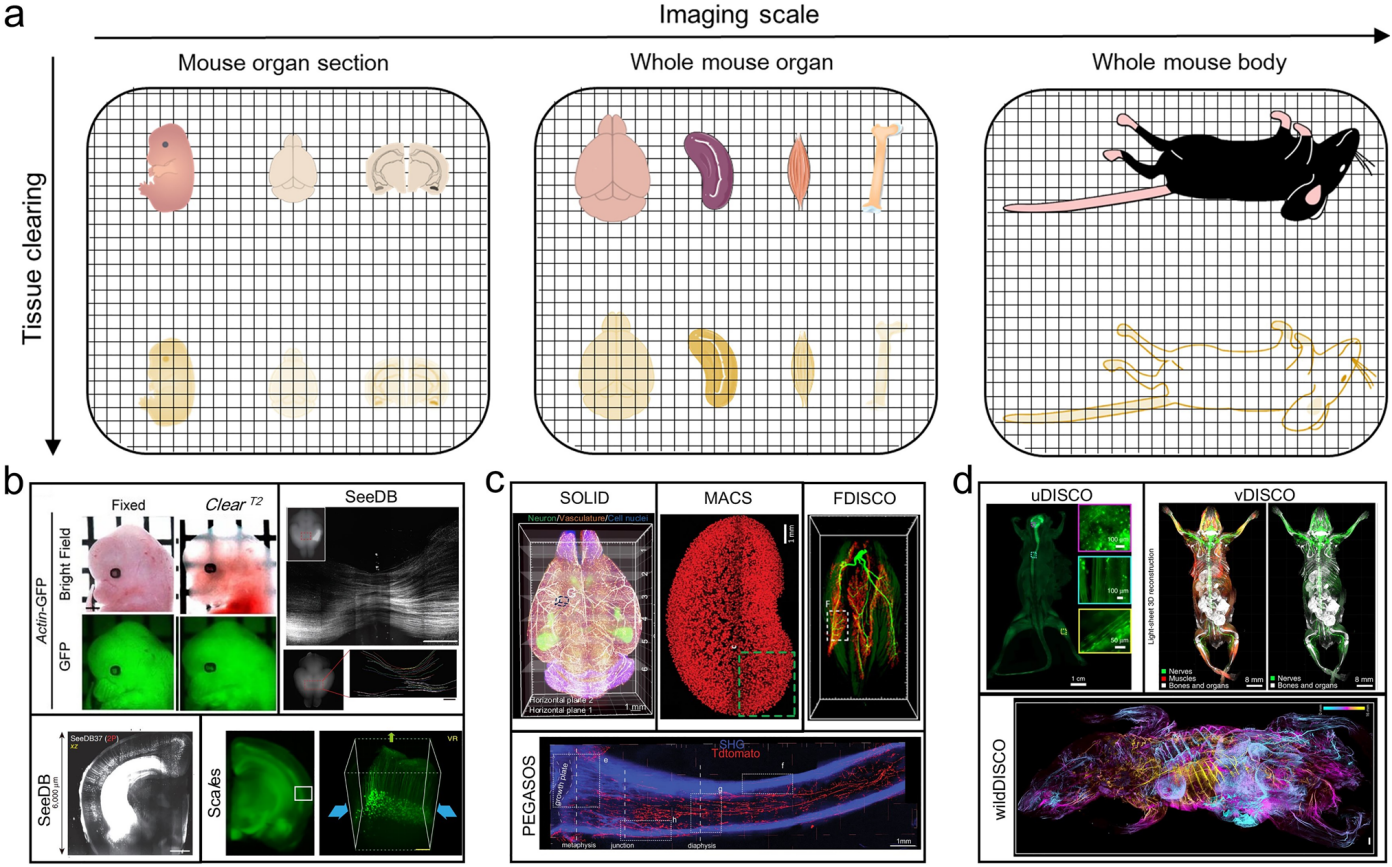
Tissue clearing techniques enable 3D imaging across multiple scales. **a** Progression of clearing methodologies from murine brain sections to small-volume specimens and ultimately human organs. **b** Small murine tissue samples were cleared using distinct protocols (*Clear*^*T2*^ [55], SeeDB [29], and Sca*l*eS [32]). **c** Whole-organ clearing of adult murine specimens was achieved through SOLID [42], MACS [52], FDISCO [36], and PEGASOS [39] techniques. **d** Whole-body clearing of adult mouse accomplished via uDISCO [35], vDISCO [56], and wildDISCO [57] approaches

The evolution of tissue optical clearing technologies over the past decade represents a remarkable history of chemical strategies progressively overcoming scale limitations. From millimeter-scale brain slices to centimeter-scale whole organs, and further to intact rodent bodies, each scale expansion has been accompanied by critical innovations in chemical formulations and penetration kinetics (Fig. 2a). Tuchin et al. systematically established the theoretical foundation in 1997 [28], which first proposed using hyperosmotic, high RI (1.38–1.50) aqueous reagents for tissue optical clearing, a principle that has guided subsequent developments. Early applications primarily focused on small mouse specimens (millimeter-scale). Ke et al. developed SeeDB (See Deep Brain) [29] and SeeDB2 [30] using fructose, though limited by high viscosity. This prompted the development of alternative methods, including FRUIT (a method based on fructose and urea) [31], Sca*l*e (an aqueous reagent that renders biological samples transparent), Sca*l*eS [32] (a sorbitol-based Sca*l*e), and 2,2ʹ-thiodiethanol (TDE)-based approaches [33] (Fig. 2b).

The field transitioned from single reagents to composite systems as research demands expanded from millimeter-scale slices to centimeter-scale whole organs. This evolution vividly demonstrates how chemical strategies, through precise molecular-level design, address the fundamental balance between large-scale penetration and structural preservation. Three major methodological branches have emerged: organic solvent-based methods, exemplified by 3DISCO (three-dimensional imaging of solvent-cleared organs) [34], achieve transparency through gradient dehydration and RI matching. Subsequent improvements include uDISCO (ultimate DISCO) [35], FDISCO (DISCO with superior fluorescence-preserving capability) [36], iDISCO (immunolabeling-enabled DISCO) [37], and iDISCO+ [38], with PEGASOS (polyethylene glycol (PEG)-associated solvent system) [39] specifically enabling mouse bone clearing. Specialized variants like sDISCO (stabilized DISCO) [40], Dec-DISCO (decolorization DISCO) [41], and SOLID (Suppressing tissue distortion based on synchronized dehydration/delipidation treatment with 1,2-hexanediol [1,2-HxD] mixtures) [42] were later developed for specific imaging needs including vascular network mapping [43–45]. Aqueous-based methods, with CUBIC (Clear unobstructed brain imaging cocktails) [46–49] as the breakthrough example, employ delipidation and RI matching. This was followed by innovative approaches like FAST 3D [50], EZ Clear [51], and MACS (the MXDA-based aqueous clearing system) [52]. Hydrogel-embedding methods represent a paradigm-shifting alternative, transforming biological tissues into hydrogels through chemical crosslinking. The landmark CLARITY [57] (clear lipid-exchanged acrylamide-hybridized rigid imaging/immunostaining/in situ hybridization-compatible tissue-hydrogel) pioneered this approach using hydrogel embedding combined with electrophoretic delipidation for whole-organ clearing. Derived methods like SWITCH (system-wide control of interaction time and kinetics of chemicals) [53] and SHIELD (Stabilization to harsh conditions via intramolecular epoxide linkages to prevent degradation) [54] have been widely adopted for combined labeling and clearing (Fig. 2c).

In the field of tissue optical clearing technology, current methodological systems each exhibit distinct characteristics. Classical organic solvent-based methods, such as 3DISCO [34], can achieve rapid tissue clearing but lead to endogenous fluorescence quenching. The improved FDISCO [36] method successfully preserves endogenous fluorescence through precise regulation of pH and temperature parameters. Yet, traditional organic solvent methods still generally suffer from the limitation of high tissue shrinkage rates. Aqueous-based methods, represented by CUBIC [46], can better maintain endogenous fluorescence signals but face challenges of prolonged processing cycles and tissue swelling. The MACS [52], through the introduction of MXDA, not only reduces the clearing time of aqueous methods but also significantly enhances compatibility with lipophilic dyes. Hydrogel-embedding methods like CLARITY [58] can achieve efficient lipid removal, but their dependence on electrophoresis equipment limits operational convenience. The newly developed SOLID [42] utilizes 1,2-hexanediol to simultaneously achieve delipidation and dehydration, resulting in minimal tissue deformation, clearing in mouse organ samples for the first time through precise “expansion–contraction” regulation. Notably, these technical optimizations currently primarily target rodent models, systematic optimization for larger animal volumes—particularly those with structural complexities like thickened myelin and dense extracellular matrices—remains challenged.

The transition from mouse organs to whole-body clearing marked a breakthrough, with the core challenge being coordinated processing of heterogeneous tissues. This advancement enabled 3D visualization of entire biological systems at single-cell resolution. Ertürk’s team reported whole-mouse body clearing using uDISCO [35], later developing vDISCO [Nanobody(V_H_H)-boosted DISCO] [59] and wildDISCO (whole-body immunolabeling-enabled DISCO) [57] for whole-body labeling via the circulatory system. Alternative approaches like HYBRiD (Hydrogel-based Reinforcement of three-dimensional imaging with chemical Dehydration) [60], iDISCO [37], iDISCO + [38], SOLID [42], and TESOS (Transparent Embedding Solvent System) [61, 62] were subsequently reported. Perfusion-based methods, including CUBIC-perfusion [63] and PARS (perfusion-assisted agent release in situ) [64], utilize the vascular system, while ACT-PRESTO (active clarity technique-pressure related efficient and stable transfer of macromolecules into organs) [65] achieves whole-body clearing through active electrophoresis. Current strategies for scaling beyond centimeter dimensions focus on: (a) optimal utilization of circulatory systems; (b) zonal processing strategies for heterogeneous tissues; (c) molecular size-tissue porosity matching designs; (d) application of external forces to enhance reagent diffusion. These approaches collectively accelerate both optical clearing and labeling processes in large-scale specimens (Fig. 2d).

## Characteristics of large animals

### Comparative analysis of tissue component and structure across species

While tissue optical clearing has been successfully applied to murine specimens at various scales, its cross-species translation faces significant challenges due to fundamental differences in tissue architecture. The large animals (e.g., pig, non-human primate, and human) exhibit distinct tissue properties (e.g., thickness [66], cellular density [67, 68], and extracellular matrix composition [69]) compared with those in large animals (e.g., pig, non-human primate, and human). These differences directly impact reagent penetration efficiency, RI matching precision, and ultimate imaging quality. Therefore, systematic evaluation of species-specific variations in myelination patterns within the nervous system and structural heterogeneity in parenchymal organs is critical for understanding the limitations of tissue optical clearing in large model animals.

#### Tissue composition variations in the brain

**Fig. 3:**
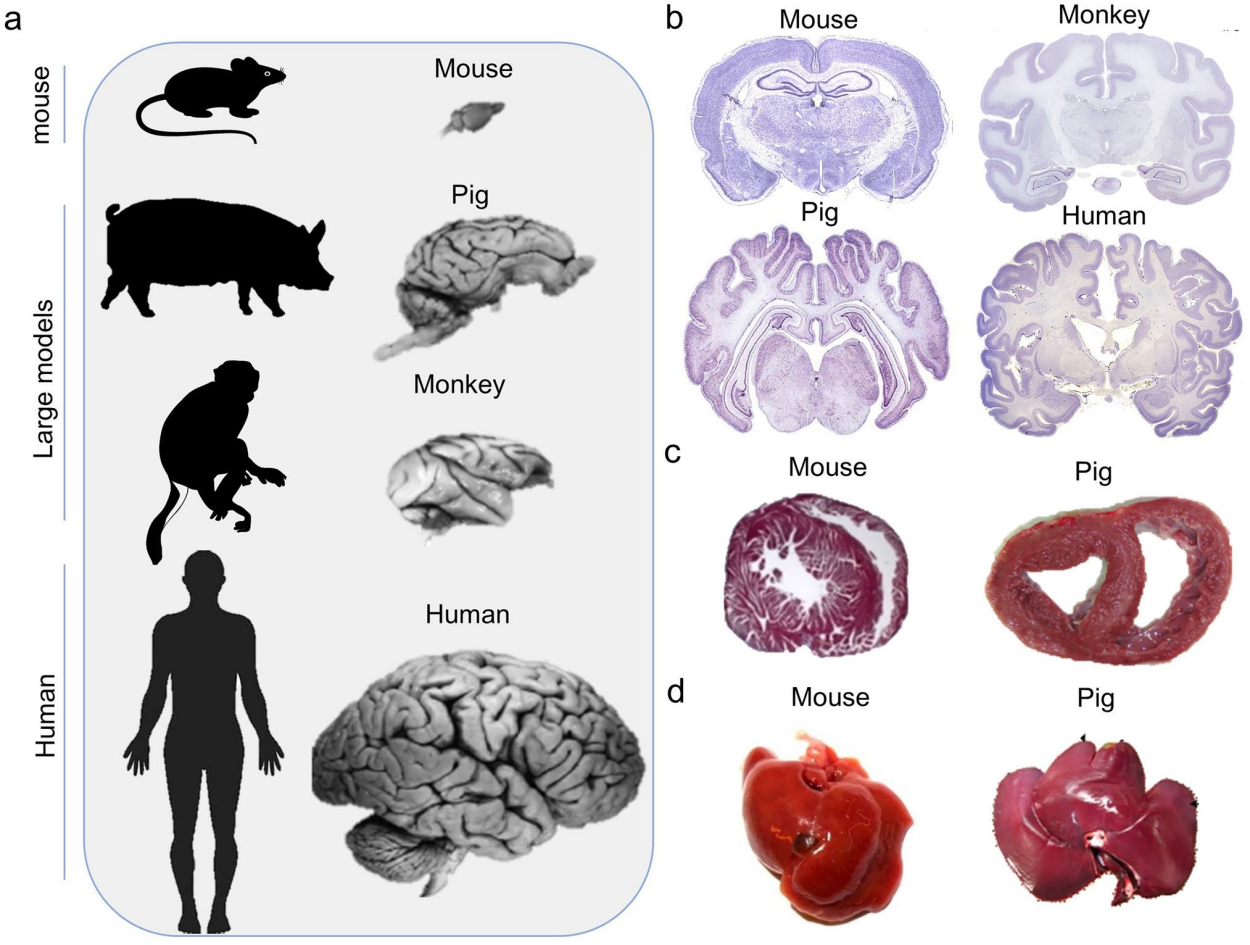
Structural differences among large model animals (pig, monkey), mouse, and human. **a** Comparative whole-brain volumes across large model animals, mouse, and human. Adapted from Yin et al. Protein & Cell [70]. **b** Variations in myelination patterns and white matter content in the brains of large model animals versus mouse and human. Mouse: Adapted from Brainmaps.org (UC Regents Davis). Pig: Adapted from Ulyanova et al. eNeuro [71]. Monkey and human: Adapted from brainmuseum.org (UW-MSU/NMHM), supported by NSF/NIH. **c** Differences in tissue density between porcine and murine cardiac tissue. Adapted from Yen and Hsieh. Front. Bioeng. Biotechnol. Conference [72]. **d** Structural and morphological distinctions between porcine and murine hepatic tissue. Adapted from Foquet et al. [73] and Kamimura et al. [74]

The brain tissues of different species exhibit significant differences in gray/white matter distribution [75], lipid content [76], myelin density [77], tissue stiffness [78], and vascularization patterns [79], demonstrating an evident evolutionary gradient from rodents to non-human primate and human (Fig. 3a and b). Regarding white/gray matter ratios, murine brains show the highest proportion of gray matter (~ 86%) [80–82], with relatively sparse white matter predominantly localized in deep regions [83, 84]. In contrast, non-human primate and porcine brains display substantially increased white matter content (~ 39%) [85, 86], featuring well-developed subcortical white matter tracts. Human brains exhibit the highest white matter proportion (~ 45%) [87], including highly specialized pathways (e.g., the arcuate fasciculus). Lipid composition and content also vary across species. Murine brains contain the lowest lipid levels, primarily phospholipids, while non-human primate brains show elevated cholesterol content [88]. Porcine brains share lipid profiles with human but exhibit higher oxidative modifications [89]. Human brains possess the highest lipid content, enriched with long-chain fatty acids, which directly influence tissue optical properties [90]. Myelin density follows an ascending trend from rodents to non-human primate [91–93]. Mouse exhibits the thinnest and most uniformly distributed myelin sheaths, whereas non-human primate display moderately thickened myelination, particularly in motor pathways. Porcine brains closely resemble human myelin characteristics, while human brains demonstrate the thickest and most regionally specialized myelination [94, 95]. Tissue mechanical properties also differ substantially. Murine brains are the softest, while non-human primate tissues show increased stiffness [96]. Porcine brains exhibit greater rigidity due to enhanced collagen deposition [97], and human brains display the highest tissue stiffness [98]. Vascularization patterns are similarly species-specific. Murine brains possess simple, low-density vascular networks [99]. Non-human primate brains develop more complex cortical vasculature, and porcine brains closely mimic human vascular distribution but with higher calcification propensity [100]. Human brains feature the most intricate vascular systems, particularly in white matter penetrating vessels [101, 102].

#### Structural heterogeneity in parenchymal organs

Parenchymal organs exhibit remarkable interspecies variations in their histological organization (Fig. 3c and d). At the cellular level, rodent organs are characterized by uniform cell arrangements with high cytoplasm-to-nucleus ratios, while non-human primate organs display greater cellular heterogeneity with prominent stromal cell components [103]. Human tissues further diverge through the accumulation of age-related pathological modifications [104]. The extracellular matrix composition shows progressive changes across species, with collagen content following a distinct porcine > human > non-human primate > rodent gradient, while elastic fiber distribution patterns demonstrate organ-specific organization [105, 106]. These structural differences are paralleled by variations in lipid profiles, where non-human primate tissues contain higher membrane cholesterol than their rodent counterparts, with each species exhibiting unique phospholipid-to-sphingolipid ratios [107]. Vascular architecture presents another layer of interspecies divergence. Large model animals generally provide closer approximations to human vascular organization compared to smaller species [19, 108]. Pigment distribution patterns further differentiate species, with non-human primate skin showing elevated melanin content and aged human tissues accumulating significant lipofuscin deposits [109, 110]. Postmortem tissue stability varies considerably, with human specimens exhibiting accelerated autolysis rates and enhanced protein cross-linking compared to animal models [111].

### Cross-species tissue clearing protocol challenges

Although tissue optical clearing techniques have achieved relative maturity in rodent models, their translation to large animals faces substantial challenges stemming from fundamental differences in tissue scale, architecture, and biochemical composition between these species. The primary obstacle arises from the dramatic increase in tissue dimensions, creating critical bottlenecks in reagent penetration efficiency. While rodent organs (e.g., mouse brains) typically achieve full reagent penetration within days, comparable concentrations may require weeks or even months to permeate large-scale specimens like porcine brains or non-human primate kidneys. This dimensional scaling effect not only significantly prolongs experimental timelines but often results in over-processed peripheral regions alongside inadequately cleared core areas. Furthermore, tissue compositional differences impose stricter requirements on clearing strategies. The central nervous systems of large animals contain substantially more abundant myelin structures, while their parenchymal organs (e.g., liver, heart) exhibit markedly higher extracellular matrix density compared to rodents. Consequently, conventional lipid-clearing agents and RI-matching solutions effective in rodents frequently prove inadequate for complete lipid removal or homogeneous tissue optical clearing in large animal tissues. The balance between structural preservation and clearing performance becomes particularly challenging at larger scales. Prolonged processing of large animal specimens increases the risk of protein antigenicity loss and structural collapse, severely compromising subsequent immunolabeling and 3D reconstruction.

Beyond these intrinsic biological constraints, the reliable application of clearing techniques in large animals remains blocked by additional methodological and technical barriers: (1) Standardization deficits: the absence of unified evaluation criteria for large animal clearing leads to substantial variability in critical parameters (e.g., permeation duration, reagent concentration) across research groups, undermining both reproducibility and systematic optimization; (2) Imaging limitations: post-clearing visualization of large organs demands advanced imaging systems (e.g., enhanced light-sheet microscopy) with greater penetration depth for whole-organ 3D reconstruction—technologies not yet widely accessible; (3) Species-specific adaptation gaps: Current protocols predominantly employ direct rodent-to-large animal translations with parameter adjustments, lacking dedicated frameworks addressing unique requirements of different large species. These multifaceted challenges collectively constitute the major obstacles in adapting tissue optical clearing technologies for large animal applications, necessitating coordinated solutions across chemical engineering, protocol standardization, and imaging innovation.

### Tissue optical clearing methods for large animal

In recent years, several studies have successfully applied tissue optical clearing techniques to large animal specimens. However, significant differences in tissue structure, size, and biochemical properties have posed considerable challenges. Current research on tissue optical clearing methods for human tissue blocks remains exploratory, with most work relying on adaptive modifications of existing rodent-optimized protocols. Several studies have directly transferred established mouse clearing protocols, such as iDISCO [112], uDISCO [35], CUBIC [49], UbasM (Urea-Based Amino-Sugar Mixture) [113], CLARITY [58], and PACT [64] to human neurological disease research by modifying incubation times. Due to the high myelin density in human brains, age-related lipofuscin accumulation, protein aggregation, and autofluorescence from residual blood in non-perfused tissues, most methods could only be effectively applied to thin sections of 100–1000 μm thickness [114]. Subsequent research combining CLARITY with alternating active and passive clearing approaches, along with extended clearing durations, achieved successful clearing of 8 mm-thick human brain sections [115]. Furthermore, studies have demonstrated that clearing efficiency varies significantly depending on species (human vs. rodent), brain region, and fixation status (fresh vs. formalin-fixed tissues) [114]. To address these challenges, several methods specifically designed for human tissue characteristics have been developed in recent years, including MASH (Multiscale Architectonic Staining of Human cortex) [116], OPTIClear (Optical properties-adjusting tissue-clearing agent) [117], hFRUIT (an optimized version of the original FRUIT) [118], and ELAST (entangled link-augmented stretchable tissue-hydrogel) [119], all capable of clearing 5–10 mm-thick human tissue blocks. Notably, the SHANEL (Small-micelle-mediated human organ efficient clearing and labeling) [120] method successfully achieved whole human organ clearing by employing CHAPS (3-[(3-Cholamidopropyl)dimethylammonio]-1-propanesulfonate), a small-micelle reagent. Several studies have developed specialized tissue clearing methods tailored for large model animal tissues, such as Sca*l*eSF [121] (a glutaraldehyde-resistant tissue clearing method), a glutaraldehyde-resistant approach that enables multi-scale light and electron microscopy integration in rodents and primates, and PuClear [122] (a primate-optimized uniform clearing method), a primate-optimized technique using Triton X-100 permeabilization with high-refractive-index matching to achieve uniform transparency in thick macaque brain slices while preserving tissue morphology.

## Advances in tissue optical clearing applications for large animals

The rapid advancement of tissue optical clearing techniques has enabled increasingly widespread applications across large animal species. In human studies, these methods have been successfully implemented for neuroanatomical mapping and disease pathology characterization, as comprehensively reviewed by Mai and Lu [25]. Building upon these foundations, this section will specifically examine the growing applications of tissue optical clearing in established large model animals. The current landscape of tissue optical clearing applications in large model animals reveals distinct patterns of implementation across different organ systems. Based on comprehensive analysis of existing literature, successful applications have primarily focused on four major categories: the central nervous system (including primate brains and spinal cords), sensory organs (such as porcine cochleae and ferret retinas), cardiopulmonary systems (encompassing primate lymph nodes and porcine lungs), and endocrine/metabolic tissues (notably porcine pancreas and primate bone marrow). This classification emerges naturally from the fundamental tissue characteristics that determine clearing efficacy—the dense myelination of neural tissue requires fundamentally different approaches than the mineralized matrices of sensory organs or the lipid-rich environments of endocrine tissues. By organizing the research according to these biologically meaningful categories, we aim to provide readers with a structured framework for understanding how clearing methodologies must be adapted for different tissue types while highlighting the most promising areas of application in large model animals. This systematic approach not only reflects the current state of the field but also serves to identify important gaps where further methodological development may be needed.

### Central nervous system research

**Fig. 4:**
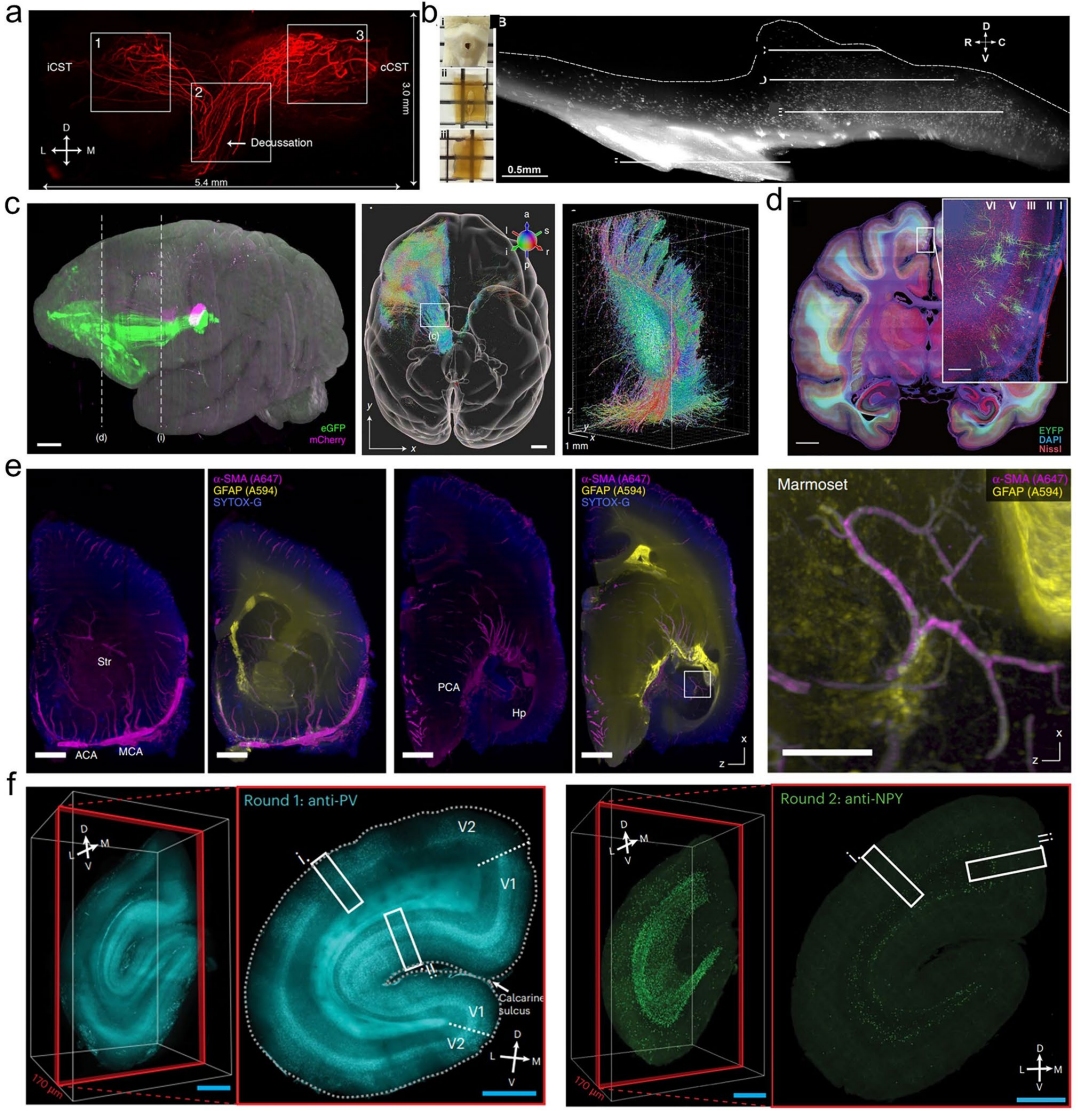
Applications of tissue optical clearing technology in central nervous system research. **a** 3D imaging of marmoset spinal cord (modified 3DISCO) [123]. **b** Sheep hypothalamic KNDy neurons (optimized iDISCO) [124]. **c** Macaque brain slice clearing (300 μm, PuClear) [122]. **d** Cynomolgus viral injection sites (PuClear) [125]. **e** Marmoset hemisphere (CUBIC-HistoVIsion) [126]. **f** Marmoset PV + /NPY + neurons (SHIELD + eFLASH) [127]

Large model animals’ brain volume and cortical gyrification patterns closely resemble those of humans, making them indispensable for studying the neural basis of higher cognitive functions. Recent developments in tissue optical clearing have provided breakthrough tools for 3D imaging of neural networks in large animal organs. Soderblom et al. employed a modified 3DISCO clearing method, AAV viral labeling, and chemical tracing to achieve high-resolution 3D imaging of non-human primate spinal cords. Their work elucidated the interaction between axonal regeneration and scar formation after spinal cord injury, establishing a versatile cross-species platform for neural regeneration research (Fig. 4a) [123]. Moore et al. adapted the iDISCO technique for sheep hypothalamic studies, achieving the first 3D visualization of KNDy (kisspeptin/neurokinin B/dynorphin) neurons, and mapping of their distribution in the arcuate nucleus and unexpectedly identified a subpopulation of kisspeptin neurons in the lateral hypothalamic area (Fig. 4b) [124]. Xu et al. developed an efficient imaging approach (SMART, semiautomated reconstruction and tracing) that integrates sequential sectioning with PuClear (a primate-optimized uniform clearing method), a clearing method based on CLARITY and CUBIC, optimized membrane permeabilization with Triton X-100 and high RI matching (RI = 1.52), overcoming penetration limitations in primate brain tissue. This enabled the first high-throughput 3D reconstruction of an entire macaque brain, revealing fine-scale thalamocortical projection patterns [122]. Subsequent application in cynomolgus monkeys achieved complete single-neuron morphological reconstructions, uncovering diversity in primary motor cortex neurons (Fig. 4c and d) [122, 125].

The CUBIC-HistoVIsion technique optimized staining parameters under electrolyte gel conditions, achieving high-uniformity labeling of astrocytes (GFAP) and blood vessels (α-SMA) in adult marmoset brain hemispheres. This provides a reliable method for glial cell studies in large animals (Fig. 4e) [126]. Furuta et al. developed the Sca*l*eSF clearing technique for 1 mm-thick marmoset cortico-striatal sections, providing a crucial tool for multiscale LM/EM connectomic analysis across mammalian brains [121]. Zhao et al.’s SHANEL leveraged small CHAPS detergent micelles for deep penetration, successfully clearing intact adult porcine brains and human brain [120]. Leuze et al. utilized the CLARITY clearing method with SWITCH immunostaining to perform imaging of fluorescently labeled neurofilaments and vasculature in occipital lobe tissue blocks from macaques. By correlating these findings with diffusion MRI (dMRI), they established a high-resolution histological validation framework for multimodal brain connectivity studies [128]. Yun et al. combined SHIELD with eFLASH [129] (electrophoretic-Fast Labeling using Affinity Sweeping in Hydrogel) to maintain dynamic chemical equilibrium while enhancing diffusion via electrophoresis. This allowed uniform immunolabeling of marmoset visual cortex blocks (5 mm × 5 mm × 8 mm), successfully resolving laminar distributions of PV + (Parvalbumin-positive) and NPY + (Neuropeptide Y-positive) neurons (Fig. 4f) [127].

### Sensory organ research

**Fig. 5:**
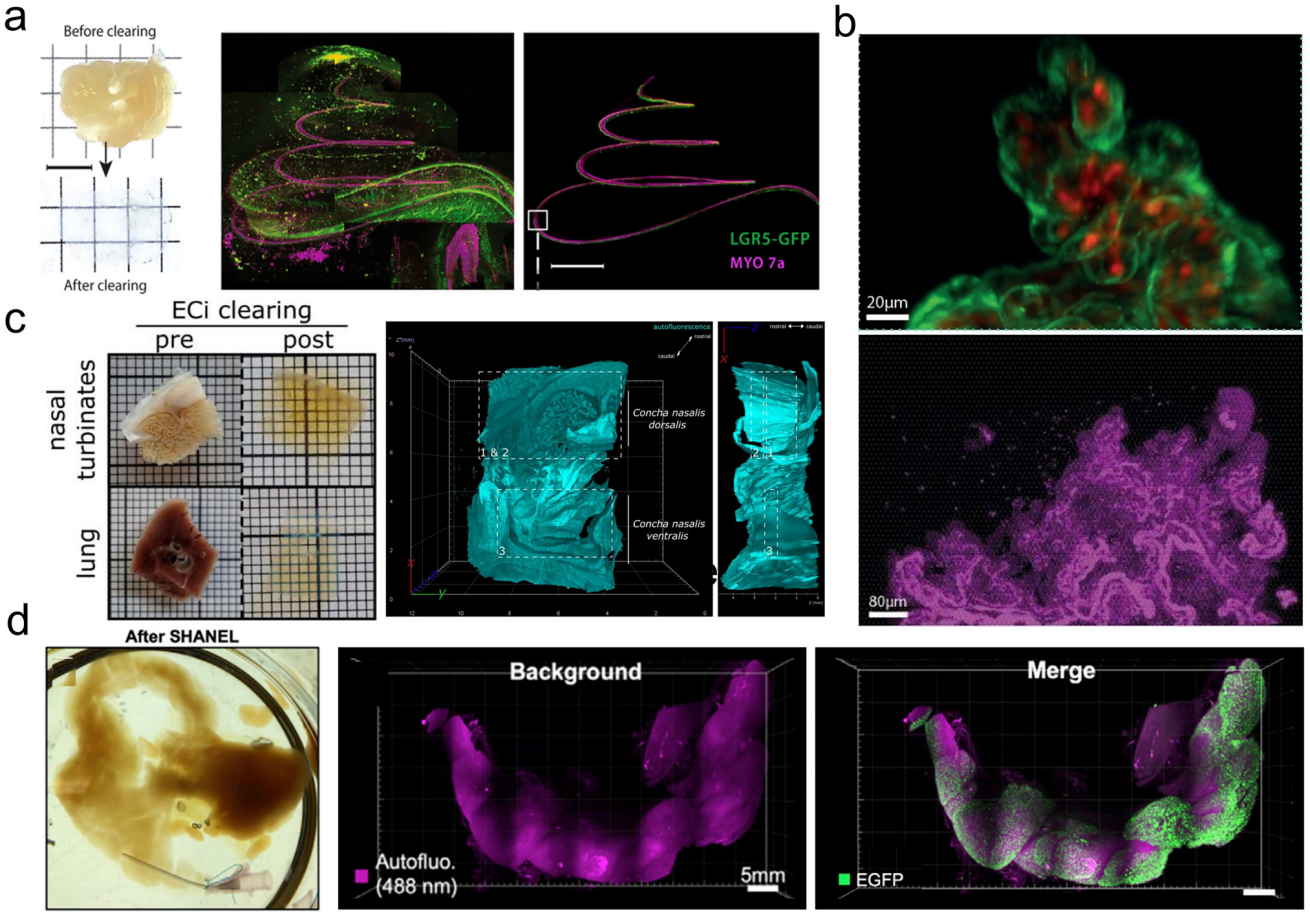
Applications of tissue optical clearing technology in other physiologic systems. **a** 3D imaging of the pig cochlear using BoneClear tissue clearing [130]. **b** Imaging of placental microvasculature and auto-fluorescence in rhesus macaques using Visikol.®-based tissue clearing [134]. **c** 3D imaging of ferret respiratory tract using ethyl cinnamate (ECi)-based tissue clearing [136]. **d** Adult porcine pancreas clearing using SHANEL [120]

Tissue optical clearing technology has also achieved remarkable progress in the visualization of sensory organs. Moatti et al. optimized the BoneClear technique to accomplish the first successful clearing of intact African green monkey cochleae, enabling high-resolution 3D imaging of hair cells and spiral ganglion neurons through light-sheet fluorescence microscopy (Fig. 5a) [130]. The same research team further adapted the clearing protocol to overcome the challenge of high bone density in large animal cochleae, achieving 3D visualization of intact porcine cochleae from neonatal to adult stages. This advancement revealed the spatial organization of hair cells and supporting cells and permitted quantitative analysis of structure–function relationships, including frequency mapping parameters, thereby providing critical technical support for auditory regeneration research [131]. In a parallel development, Ye et al. combined CUBIC-based clearing with hydrogen peroxide bleaching to achieve the first transparency of intact ferret eyeballs, allowing single-cell resolution visualization of specific retinal cells and structures throughout the entire eye. This breakthrough established a whole-eye scale analytical tool for investigating ocular diseases in large model animals [132].

### Respiratory and circulatory system research

As an extended support system of the central nervous system, 3D imaging of respiratory and circulatory organs is crucial for understanding holistic physiologic mechanisms. Martin et al. employed a hybrid protocol combining iDISCO and CUBIC methods to clear and image lymph nodes (LNs) in rhesus macaques, revealing the precise localization of vaccine components within LNs and elucidating the mechanisms of nanoparticle antigen targeting to follicles. This work provides fundamental evidence for designing efficient lymph-targeting vaccines [133]. Sargent et al. developed an optimized Visikol®-based tissue clearing protocol combined with immunofluorescence labeling and confocal microscopy, enabling high-resolution 3D visualization and quantitative analysis of the placental microvasculature in nonhuman primates, providing a powerful tool for investigating placental microanatomy in pregnancy-related pathologies (Fig. 5b) [134]. Schwenninger et al. systematically compared the effects of multiple tissue optical clearing agents on porcine lung tissue biomechanics. The tested agents included dimethyl sulfoxide (DMSO), aniline mixtures, and benzyl alcohol/benzyl benzoate (BABB), among others. They identified that a 1:1 mixture of DMSO and aniline achieved optimal tissue clearing while best preserving the native biomechanical properties of porcine tissue, offering important methodological references for 3D organ imaging [135]. Zaeck et al. implemented ethyl cinnamate (ECi)-based clearing method to achieve 3D visualization of SARS-CoV-2 infection in ferret respiratory tracts, uncovering the oligofocal infection pattern of the virus. Comparative analysis with traditional histology highlighted the unique advantages of this method in resolving spatial organ architecture, establishing new technical pathways for developmental biology studies (Fig. 5c) [136]. Kim et al. successfully adapted Pocket CLARITY (a passive CLARITY technique) for porcine heart studies. Through 3D imaging of healthy, heart failure, and myocardial infarction tissues, they demonstrated significant degeneration of myocardial helical structures under pathological conditions [137]. Susaki et al. developed CUBIC-HistoVIsion and achieved whole-body 3D staining and imaging in infant marmosets, revealing interspecies differences in vascular and glial cell distribution patterns [126].

### Endocrine and metabolic system research

The 3D structural analysis of the endocrine system, which is closely interconnected with neural and circulatory systems, has provided novel perspectives for metabolic disease research. Theobalt et al. employed 3DISCO method combined with light-sheet microscopy to achieve 3D quantitative analysis of adipose tissue in obese porcine models. This work marked the first successful precise measurement of cellular volume and quantity distribution across different fat depots in large animals, revealing distinct growth patterns between visceral and subcutaneous adipocytes [138]. Robino et al. successfully overcame light scattering issues caused by lipids in macaque bone marrow using Visikol® tissue clearing reagent, enabling clear 3D resolution of the spatial relationship between hematopoietic stem cells and bone marrow adipose tissue [139]. SHANEL has also been successfully applied for 3D imaging of transgenic porcine pancreas to visualize the spatial distribution of pancreatic β-cells, establishing an organ-scale analytical tool for investigating mechanisms of metabolic diseases such as diabetes in large model animals (Fig. 5d) [120].

## Future perspectives

In the field of tissue optical clearing technology, future development should focus on three critical areas for advancement. The first involves the refinement of sample pretreatment protocols. Unlike mouse specimens that can be effectively fixed through PFA perfusion or overnight immersion, studies on human tissues have demonstrated that a staged, progressive processing approach yields superior results, requiring dynamic adjustment of reagent concentrations and processing durations based on tissue response characteristics to better preserve structural integrity. The second key area is the enhancement of penetration efficiency. To address the limitations of simple immersion techniques for large specimens, passive diffusion strategies can incorporate approaches like the SHANEL [120], which utilizes small-micelle zwitterionic detergents (e.g., CHAPS) to improve lipid removal and penetration. Future efforts should prioritize optimization of chemical reagent parameters [140], including micelle size and viscosity, along with physical conditions such as temperature and pH. For active diffusion enhancement, electric field-assisted technologies like SHIELD [54] and eFLASH [129] show promise, alongside the development of multimodal physical permeation strategies incorporating perfusion, electromagnetic, and ultrasonic approaches, while fully leveraging vascular networks for distributed reagent delivery. The third crucial direction is the optimization of cross-species compatibility, which demands a comprehensive, multi-tiered solution framework. This includes establishing species-specific databases containing key parameters like organ dimensions and lipid composition profiles, developing machine learning-based predictive models, designing modular reagent systems for fixation, delipidation, and RI matching, creating adaptive control technologies with real-time monitoring capabilities, and implementing standardized multi-species validation platforms with quantitative evaluation metrics such as species-specific tissue optical clearing efficiency coefficients. These synergistic innovations will substantially improve the technology’s versatility and provide vital technical support for biomedical research.

The successful application of tissue optical clearing technology in large animal model studies requires an integrated, multi-technology optimization framework. Breakthroughs in labeling techniques are particularly crucial, as current methods face two major challenges: inadequate antibody penetration into centimeter-scale sample cores and rapid decline in labeling efficiency with increasing depth. In the future, the labeling for large volumes can integrate strategies involving molecular engineering, physical enhancement approaches, as well as chemical biology strategies. For instance, creating nanobody [141] fragments with zwitterionic modifications can significantly enhance penetration while reducing nonspecific binding, employing electric field assistance and ultrasound microbubble delivery systems to improve antibody transport efficiency [142], combining cascade amplification labeling systems with metabolic precursor-based click chemistry [143, 144] to address deep-layer signal attenuation and whole-organ labeling challenges.

For imaging technology, light-sheet fluorescence microscopy remains a good alternative for large-volume specimen imaging and requires continuous advancement. Imaging volume expansion necessitates improvements in optical systems to support larger-scale comprehensive imaging [145]. Throughput enhancement demands the integration of high-speed scanning with adaptive sampling technology. Data processing efficiency can be upgraded through novel compression algorithms and storage formats [146]. Particularly noteworthy is the emergence of multimodal imaging fusion technology, which combines the high-throughput capabilities of light-sheet microscopy with the deep imaging advantages of optical coherence tomography, achieving transformative improvements in both imaging quality and efficiency [147]. Concurrently, incorporating artificial intelligence (AI) technologies [148] like deep learning [149] has revolutionized the segmentation and analysis of massive 3D image data sets, delivering remarkable advances in accuracy and processing speed.

The above collaborative technological solutions are rapidly evolving to systematically overcome the current limitations of tissue optical clearing in large animal research. Notably, while current in vivo tissue optical clearing implementations have been primarily confined to murine systems (e.g., skull optical clearing window [150, 151] and skin optical clearing [152, 153]), the ongoing development of reversible clearing methodologies combined with minimally invasive monitoring systems [154, 155] is anticipated to facilitate translational applications in large-animal models. With continued interdisciplinary cooperation, tissue optical clearing and imaging technologies are poised to deliver even greater value in life science investigations involving large model animals. The convergence of these advancements promises to bridge critical gaps between basic research and clinical applications, ultimately enhancing our understanding of complex biological systems at scales that more closely approximate human physiology and pathology.

## Conclusion

Tissue optical clearing has revolutionized 3D imaging by enabling high-resolution visualization of intact tissues, with significant progress in adapting these techniques for large model animals. This review highlights the critical interplay between methodological advancements and biological scale; successful translation from rodents to larger species requires more than simple parameter adjustments, but rather a fundamental rethinking of reagent chemistry, penetration dynamics, and tissue preservation strategies. While challenges such as tissue heterogeneity, prolonged processing times, and imaging limitations persist, recent innovations like SHANEL, CUBIC-HistoVIsion, and HYBRID protocols demonstrate feasibility for organs and systems in pig and non-human primate. The true potential of this technology lies in its ability to bridge preclinical and clinical research, offering insights into human-like physiology and disease mechanisms. However, widespread adoption will depend on standardized protocols, scalable imaging solutions, and interdisciplinary collaboration. By addressing these gaps, optical clearing can transform our understanding of complex biological systems and accelerate translational discoveries. The journey from mouse brains to human organs is not merely a technical challenge but an opportunity to redefine the boundaries of 3D structural biology.

## Data Availability

All data discussed in this review are derived from the cited references. Additional supporting information can be provided by the corresponding author upon reasonable request (dawnzh@mail.hust.edu.cn).
